# Perspective on nanochannels as cellular mediators in different disease conditions

**DOI:** 10.1186/s12964-018-0281-7

**Published:** 2018-11-08

**Authors:** Eshna Jash, Peeyush Prasad, Naveen Kumar, Taruna Sharma, Aaron Goldman, Seema Sehrawat

**Affiliations:** 1 Brain Metastasis and NeuroVascular Disease Modeling Lab, Department of Life Sciences, School of Natural Sciences, Shiv Nadar University, NCR, India; 2 Mitra Biotech, Integrative Immuno-Oncology Center, Woburn, MA 01801 USA; 3 Department of Medicine, Harvard Medical School, Boston, MA 02115 USA; 4 Division of Engineering in Medicine, Brigham and Women’s Hospital, Boston, MA 02115 USA

**Keywords:** Tunneling nanotubes (TNTs), Tumor-endothelial interaction, Tumor-immune cell interaction, Tumor-macrophages cell-cell communication

## Abstract

Tunnelling nanotubes (TNTs), also known as membrane nanochannels, are actin-based structures that facilitate cytoplasmic connections for rapid intercellular transfer of signals, organelles and membrane components. These dynamic TNTs can form de novo in animal cells and establish complex intercellular networks between distant cells up to 150 μm apart. Within the last decade, TNTs have been discovered in different cell types including tumor cells, macrophages, monocytes, endothelial cells and T cells. It has also been further elucidated that these nanotubes play a vital role in diseased conditions such as cancer, where TNT formation occurs at a higher pace and is used for rapid intercellular modulation of chemo-resistance. Viruses such as HIV, HSV and prions also hijack the existing TNT connections between host cells for rapid transmission and evasion of the host immune responses. The following review aims to describe the heterogeneity of TNTs, their role in different tissues and disease conditions in order to enhance our understanding on how these nanotubes can be used as a target for therapies.

## Background

Tunnelling nanotubes (TNTs) are intercellular cytoplasmic channels that enable direct physical interactions, facilitating the transport of cellular cargo between a range of different cell types. These have been referred to in recent literature by a number of names including “nanochannels”, “cytoplasmic extensions”, “intercellular bridges”, “nanotubular highways” or “epithelial bridges”. These are generally fine, long, non-adherent and are composed of F-actin-based extensions and function to promote intercellular cargo transfer between proximal and distant cells [1]. Generally, there are two important types of nanotube: close-ended and open-ended tunnelling nanotube. Junctional borders are present in close ended cell-to-cell bridges i.e., innate nanotubes which connect T-cells and the MLV-induces cellular bridges. Through these cellular bridge cargo has to cross the plasma membrane boundary. According to this finding HIV-1 fusion inhibitor T20 can block the receptor-dependent transmission of HIV-1 Gag [2]. On the other hand, tunneling nanotubes (TNTs) are product of de novo formation of membranous nanotubes between cells which helps in cell-to-cell communication. TNTs are F-actin rich structures which is involved in mediating membrane continuity. It is also shown to be involved in intercellular transport of various cellular components [3]. TNTs are involved in several diseases such as cancer, viral infection, Parkinson’s disease [4–6].

While similar actin-based extensions such as lamellipodia, invadopodia and filopodia exist, they differ in their properties such as diameter, adherence and cell-cell cytoplasmic interaction [7]. Furthermore, TNTs have been observed to be not affected by trypsin digestion and are devoid of microtubules [7]. The walls of the nanotubes are continuous with the plasma membrane and are hence composed of the same bilipid membrane layer and the lumen of the nanotube connects the cytoplasm of the connecting cells.

Studies have demonstrated the transfer of everything from prions [8, 9], retroviruses [10, 11], apoptotic signals [12], calcium signals [13] to full length RNA, proteins and organelles [14] through these nanochannels. Data from studies in in vitro and ex vivo experiments suggest that uninfected healthy cells have a minimal number of nanotube connections with adjacent cells, but they were observed to be significantly higher in disease conditions such as cancer, viral infections and prion-associated diseases, among others [8, 9, 15]. There are several ways such as cell-cell communication, organelle exchange, cargo delivery and tissue repair in which TNTs play important role. Using pharmacological modulator several pathological pathways can be targeted for disease management. Understanding morphology and structural organization of TNTs can help us in finding the key molecular target for treating disease. Usually TNTs are involved in immune responses. Several study suggest the localization and transfer of class 1 MHC through TNTs. To escape the immune cells, viruses usually spread from cell-to-cell through TNTs. It has been observed that Human T-cell Leukemia virus migrates from infected to uninfected cells through TNTs and avoid recognition by immune cells. It has been found that HTLV-1 protein p8 increases the viral transmission. Treatment of MT-2 cells by cytarabine reduced TNTs formation induced by HTLV-1 p8 protein. Targeting these nanotubes can aid in treating disease caused by this virus. TNTs are also found to be involved in lung adenocarcinoma tumor cells [1, 16]. Increased number of TNT formation has been observed in co-culture of rat primary astrocytes and C6 glioma cells due to oxidative stress [17]. There are several diseases associated with oxidative stress such as diabetes, Parkinson’s and cancer. In stress condition TNTs can transfer mitochondria which serves as the rescue mechanism [18].

This review highlights the functional role of TNTs in cancer development, progression and metastasis, along with its role in viral infection.

## TNTs facilitate cell-cell communication in cancer cells

These nanotubes play a vital role in the intercellular communication in cancer cells and have been characterized in multiple cancer subtypes as a facilitator of cancer cell to cancer cell communication, as well as cancer cell to tumor matrix communication. It has been postulated that this intercellular communication with the tumor matrix may play a direct role in the progression and development of solid tumors [19]. TNTs have been detected to play a role in multiple cancer types including lung adenocarcinomas [1], prostate cancer [7, 14], colon cancer [20] and glioblastoma [21].

Nanochannels between cancer cells were first described in 2004 paper using imaging techniques such as SEM (scanning electron microscopy) and TEM (transmission electron microscopy) where organelle transport was evidenced in HEK, NRK and PC12 cell lines [7]. A study in 2012 on the role of nanochannels using immunofluorescence imaging and confocal microscopy in pleural mesothelioma cells provided evidence that suggested direct bidirectional transfer of not only chemical messengers, but also proteins and organelles such as mitochondria and Golgi vesicles [1]. In this study, TNTs formed by migrating cancer cells were observed in time-lapse confocal imaging and were found to extend nanotubes during migration to fill the gaps created during a scratch wound assay which indicates a role of nanochannels in cancer invasion [1]. This is supported by the discovery of fascin, an actin filament bundling protein that has been identified as a component of nanotubes and has been previously implicated in the initiation of metastases [1].

One study identified these nanochannels as facilitators of cross-talk between tumor cells and endothelial cells during the process of metastasis. The cancer cells were observed to regulate the endothelium through the transfer of microRNAs, which was evidenced by a higher expression of pathogenic endothelium markers that was reversed upon pharmacological inhibition of nanochannel formation [22]. The research suggested that cancer cells are able to dynamically modulate healthy cells through horizontal communication using nanochannel conduits. TNTs have also been studied in prostate cancer cells where it was demonstrated through immunofluorescence imaging that alpha-tubulin also acts as a microtubule component in nanotubes that are more than 1 μ in diameter [14]. Microtubule is one of the structure which has been seen in TNTs and is suggested by several research group. TNTs are transient structures which last from few minutes to hours. Study done by Differential interference contrast (DIC) imaging found that hippocampal neurons from E18 rats make TNTs in which these transient structures have microtubules but lack F-actin [23]. Davis et al. demonstrated that macrophages form different types of TNTs which contain microtubules and F-actin. These TNTs are involved in large material transport such as lysosomes and mitochondria [24]. Here, we believe that component of TNTs are based on cell type and the function it performs during cell-to-cell communication [25]. However, there is need for identifying exact component of cell-specific TNTs.

A study on nanotubular membrane extensions in colon cancer concluded that these TNTs are extremely sensitive to mechanical stress, but also very dynamic in nature. They have the ability to retract and form channels again through extension of filopodia-like structures that make contact with the neighbouring cells [20]. The elastic properties of cancerous TNTs were further assessed on glioblastoma cells where existing nanotubes between cancer cells were observed under lateral tension and force. It was found that these nanochannels are highly elastic, and were observed to bifurcate instead of breaking when stress was applied at one point indicating much higher resilience than expected [4].

Studies have also demonstrated that malignancy can be induced by cancer cells in neighbouring healthy cells using nanochannels through the transfer of microRNAs between them. TNTs were observed to transport oncogenic microRNAs which have been identified to be upregulated in cancer, specifically miR-19a in osteosarcoma and miR-199 in ovarian cancer, to non-malignant cells [26]. Exosomes, another form of cell-cell communication, have been observe to co-localise near existing nanochannels and stimulate an increased rate of TNT formation [27].

The propagation of chemo-resistance through nanotubes between cancer cells is a compelling biological phenomenon that has been observed in few studies. This hypothesis of chemo-resistance development by cancer cells through intercellular transfer was first put forward in 1991 in Frankfurt et al., but TNTs as conduits were not implicated until much later [28]. The formation of nanochannel structures between cancer cells have been widely studied and seems to rely on the mTOR pathway, evidenced through the marked decrease in TNT formation upon suppressing the mTOR signalling by pharmacological inhibitors such as Metformin and Everolimus. This study was performed on different ovarian cancer cell lines, and also identified hypoxic microenvironment conditions as a catalyst for initiation of TNT formation. Hypoxia induces the cellular proliferation differently in chemoresistant (C200 and SKOV3) ovarian cancer cells and chemosensitive (A2780) cancer cells. Chemoresistant cellular model showed increased TNT formation and TNT mediated communication under hypoxic condition. Study found that chemoresistant SKOV3 cells showed increase in TNT numbers by 72 h with respect to normoxic conditions. This suggests TNTs might be involved in giving drug resistance to ovarian cancer cells [29].

**Fig. 1:**
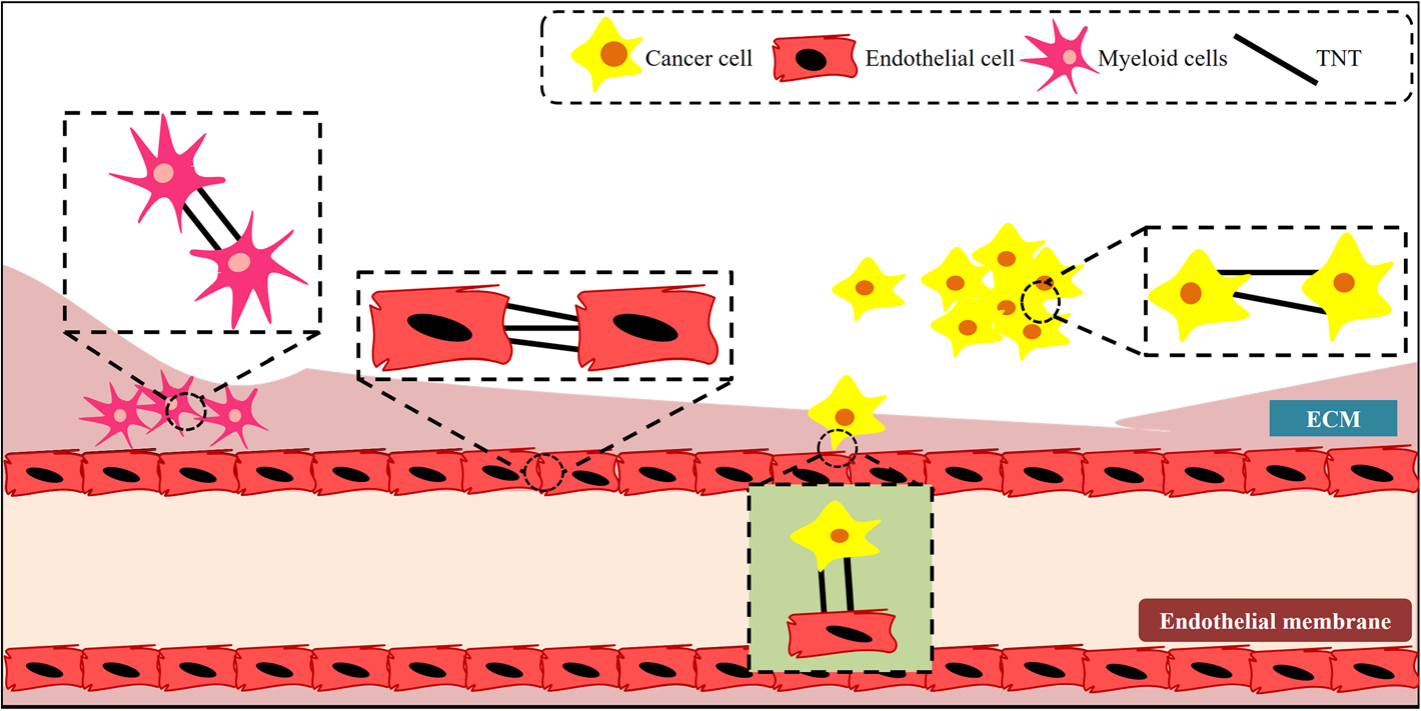
Schematic representation depicts inter and intra cellular communication between different cells using TNT’s. These TNT’s are observed to form between different cell types such as myeloid cells and cancer cells. They are also known to be involved in cancer migration and metastasis [19, 55, 44, 56]

Study published in 2013 further investigated the role of nanochannels between cancer and stromal cells in chemo-resistance cancer. They studied the TNTs formed in vitro between anchorage dependent spheroids as well as tumor explant cultures. In particular, they highlighted the transport of mitochondria through nanotubes between endothelial and cancer cells (ovarian and breast cancer cells) that corresponded with an acquired chemo-resistance in the recipient cancer cells [30]. The study also described nanotubes formed between mesenchymal stem cells and cancer cells, with similar mitochondrial transfer. Thayanithy et al. also directly observed the intercellular transfer of miRNAs between cancer cells (osteosarcoma and ovarian cancer cell lines), including those that are implicated in conferring chemo-resistance [26]. Figure 1 summarizes the different types of cell-cell communication mediated at nano-level interaction.

## Viral hijacking of host cell TNTs

Membrane nanochannels have been directly observed to be present between a variety of cell types within our body. Recent research has also identified the key role played by nanochannels during viral infections [31–33]. Studies show that viral particles are capable of utilising the physical conduits between infected and non-infected cells in order to spread faster and infect a larger number of host cells. Viral transmission through intercellular conduits such as TNTs is one of the mechanism for virus particles to overcome the rate-limiting step of cell infection by diffusion through the matrix. Travelling through cells also ensures their survival by providing protection from antibodies and complement proteins in the extra-cellular serum. HTLV-1 virus escape from immune recognition by escaping through intercellular transmission [34]. There can be several mode for viral transmission from cell-to-cell. Here, we want to highlight the fact based on few studies that cell-to-cell transmission through TNTs is one of the rate limiting step. In general, transmission through TNTs is one of the ways viruses escape from recognition by immune system [34]. Study on gap junction (GJ) channels (Cx43) and TNTs showed that these two structures are important for efficient cell-to-cell communication and viral spread. Study also identified Cx43 protein localization in intracellular compartment at the base of the TNT. Study concluded that in macrophage Cx43 expresses and localized into the TNT in the presence of HIV infection [35]. Another study found the presence of HIV particles within the TNTs and proposed that HIV highjacks the TNT communication for spreading from cell-to-cell [15]. Despite of these evidences more researches are required in this area.

According to a study published in Nature Cell Biology [2], HIV transmission through physical intercellular connections is 100–1000 times more efficient than cell-free transmission. Membrane nanotubes between T cells are formed when they come in contact with one another, and the transmission of HIV viral particles through the T cell nanochannels was found to be dependent on CD4 and Env. HIV transmission was monitored through GFP-tagged Gag viral protein, and the rate of transmission through nanochannels was determined to be approximately 0.03 μm/s. This implies that TNTs are a major pathway for the transmission of HIV, and identifies them as a possible target for therapeutic strategies against HIV [2].

Another paper published also implicated the Env viral protein in playing a key role in the process of filopodial-bridge between an infected and an uninfected target cell [36]. The viral envelope glycoprotein (Env) is involved in the recognition of receptors on uninfected target cells, following which virus moves along the outer surface of filopodial-bridge towards target cells [37]. HIV infection in macrophages has also been observed to increase the initiation of nanotube formation, following which the viral particles hijack the nanotubes for efficient cell to cell transmission. The study also demonstrated that the formation of nanotubes corresponds with the time course of HIV viral replication [33].

In addition, Herpes Simplex Virus (HSV) was also observed to spread through thin intercellular cytoplasmic bridges up to 100 μm long, whose physical characteristics resemble TNTs. The study tagged an essential viral protein VP16 with a GFP tag and tracked the transmission of the viral particles through nanotubes, going from infected cells toward adjacent target uninfected host cells [38]. A study on conserved *alphaherpesvirinae* proteins also revealed that the viruses in question are able to induce cytoskeletal rearrangements to form TNTs which they subsequently used for intercellular transmission. This viral subfamily includes the herpes simplex virus (HSV), varicella-zoster virus (VZV), porcine pseudorabies virus as well as other important animal virus that are responsible for a number of diseases. Intercellular viral spread was monitored in the presence and absence of nanochannel inhibitors and this pathway for transmission was determined to enhance the spread of the virus significantly [39].

A study in 2015 on Influenza A determined that the viral proteins could be transmitted through intercellular TNTs [40]. Neuraminidase inhibitors failed to inhibit the viral spread to uninfected cells, however neuraminidase in conjunction with microtubule inhibiting drugs such as Paclitaxel had a significant impact on viral infection in vitro [15]*.*

A recent study published in 2017 also highlights the role of TNTs in the spread of influenza virus. Lung epithelial cells are physically connected to each other through extensive nanochannel networks, which was exploited by the influenza virus for efficient cell-cell transmission [33]. Confocal and scanning electron microscopy was also used to observe TNT formation before viral infection and viral transmission after infection. In the study, the viral transmission was not affected by neutralising antibodies or antiviral drugs and it was also determined that the networks could be used for complete viral genome and protein transmission [40]. In the presence of neutralizing antibodies viral genome transfer through actin-based TNT structures [33]. In the presence of antiviral drug, Zanamivir, genome transfer happens through TNTs from infected to naïve cells [41]. Study using the inhibitor such as Nocodazole (microtubule inhibitor) found that TNT formation was attenuated in A549 cells. This also affected the cell-to-cell spread of viral genome [33]. In Table 1 we have summarized the important development in this field.

**Table 1:** Summary of significant reports in the field of TNT biology

Research	Findings	References
Discovery	Protrusion based communication which includes TNTs are ubiquitous. They were reported clearly for the first time by Rustom et al. in rat neuronal PC12 cells	[7, 44, 55]
Structural composition	Different types of TNTs have differences in the cytoskeleton and lipid composition. Thin membrane nanotubes contain only F-actin whereas thick membrane (> ~ 0.7 μm diameter) contain F-actin as well as microtubules. Presence of specific organelles in different types of TNT’s were reported. Thick membrane nanotubes contain mitochondria, late endosomes, lysosomes and intracellular vesicles unlike thin membrane nanotubes.	[24]
Characteristic properties	Radii in between 25 and 100 μm; form connections between cells; cytoskeleton proteins such as F actin and microtubules are present; help in propagation of selected molecules and vesicles between cells; sensitive to mechanical stress; formed through de novo actin driven protrusions as well as through alternative mechanisms.	[21, 44]
Function	• Significant transfer of cell surface protein and mitochondria in between cells through TNT’s was reported. • TNTs are found in myeloid cells where they perform different functions important for their cellular communication. • Calcium fluxes through nanotubes which has role in cellular communication. • Role of TNTs in senescent endothelial cell rescue. • Transport and hijacking of TNTs by prions.	[8, 9, 12, 52, 44, 57–59]
Role in disease progression	• HIV transmission through TNTs between T cells, Influenza virus transmission through TNTs, TNTs formation in HIV infected cells (macrophages), TNTs are also found during the progression of HSV and PRRSV infection. • Modulation of chemo-resistance in cancer through endothelial to cancer cell mitochondrial transfer. Modulation of endothelial phenotype through cancer TNTs, Induction of TNT formation in ovarian cancer cells through hypoxia conditions, Involvement of TNTs in tumor growth, differentiation and resistance to therapies. • Transfer of oncogenic mRNAs by TNTs; Induction of TNTs by tumor exosomes. • TNTs in ischemic stroke recovery. • Role of TNFα-induced protein 2 (TNFAIP2) in TNT formation	[2, 6, 10, 11, 22, 26, 27, 29, 30, 32, 39, 40, 47, 53, 60–63]

## TNTs mediate intercellular communication between immune cells

TNTs play a very big role in the cellular communication between a number of cell types in the human body, but nanochannel communications between cells of the immune system in particular have been widely studied in recent times [3, 24, 42, 43]. Nanotubular highways between different immune cells was first characterised by Önfelt et al. [44], and found TNTs between human peripheral blood NK cells, macrophages, and EBV-transformed B cells. According to the study, nanochannels can be created as immunological synapse move apart. They used single photon-excitation resonance scanning confocal microscopy to observe nanotubes in vitro between immune cells. Using GFP-tagged proteins, they also determined that cell surface proteins could be transferred to different cells through nanotubes over tens of microns and Class 1 MHC molecules were found to localise in these structures [44]. Nanotubular networks can arise from transient intercellular contacts between immune cells. Time-lapse imaging showed the formation of long nanotube connecting the cells over 140 μm in peripheral blood NK cells and 721.21 transfectant cells expressing GFP-tagged HLA-C. Study found that average length of nanotube in all intercellular connection was 30 μm with dynamic change in length as cells moved apart and breakage of nanotube after few minutes (4 min). It has been found that in murine macrophage cells (J774) nanotube structure breaks after sometime which led to nanotubular connection between two cells only. According to the time-lapse experiment shown by Onfelt et al., class 1 MHC was seen on the nanotube which grew with a speed of around 0.2 μm/s and lasted for 15 min. These data suggests that class 1 MHC might be present on the tip of close-ended nanotubes. Another study in this line showed the transfer of class 1 MHC through TNTs in HeLa cells and blocking actin polymerization led to the reduced HLA-A2 transfer [45].

Multiple immune cells can be connected simultaneously by these TNTs, which in turn establishes a complex intercellular network between immune cells. The same group published another paper describing the two distinct types of nanotubes that were formed between macrophages based on their physical properties [24]. Thin nanotubes were less than 0.7 μm in diameter, composed entirely of F-actin and bacteria could be trapped and transported within them, thick nanotubes were more than 0.7 μm in diameter and were comprised of both F-actin and microtubules. Thicker TNTs between macrophages were used by the cells for the transport of intracellular organelles such as mitochondria and lysosomes. It was also determined that transport through the nanochannel requires ATP as it was inhibited by the addition of azide [24]. Active transport happens through TNTs and depletion of ATP leads to decreased intercellular transfer of vesicles [3]. Transport of endocytic vesicles through TNTs are actomyosin-dependent. Study on PC12 and NRK cells showed that there is unidirectional movement of vesicles with a speed in the range of actin-dependent transport [3, 7]. Report shows that reduction in ATP production in mitochondria, activation of AMPK and inhibition of gluconeogenesis by metformin affects the TNTs [19]. It has been shown that F-actin depolymerization drugs and depletion of ATP results in complete inhibition of organelle transfer [3, 24, 46].

Multiple studies also determined the relationship between the specific types of TNTs with directionality. Thin nanotubes containing only actin as the structural component were observed to have unidirectional transfer of intracellular cargo, whereas thicker nanotubes with both actin and microtubule elements as structural components were observed to have bi-directional transfer [47]. Another study found nanotube bridges between cancerous monocytic cells, with as many as 75 TNTs per cell in areas with cells in close proximity [48]. In monocytes and dendritic cells, the nanochannels were used to transport calcium fluxes instantly from cell to cell. In addition, GFP linked proteins were shown to be able to travel between B cells through nanochannels, showing that the plasma membrane between B cells are continuous. Myeloid cells therefore have been shown to form extensive TNTs for propagation of intercellular signals, however non-myeloid cells such as fibroblasts do not exhibit similar properties [13].

**Fig. 2:**
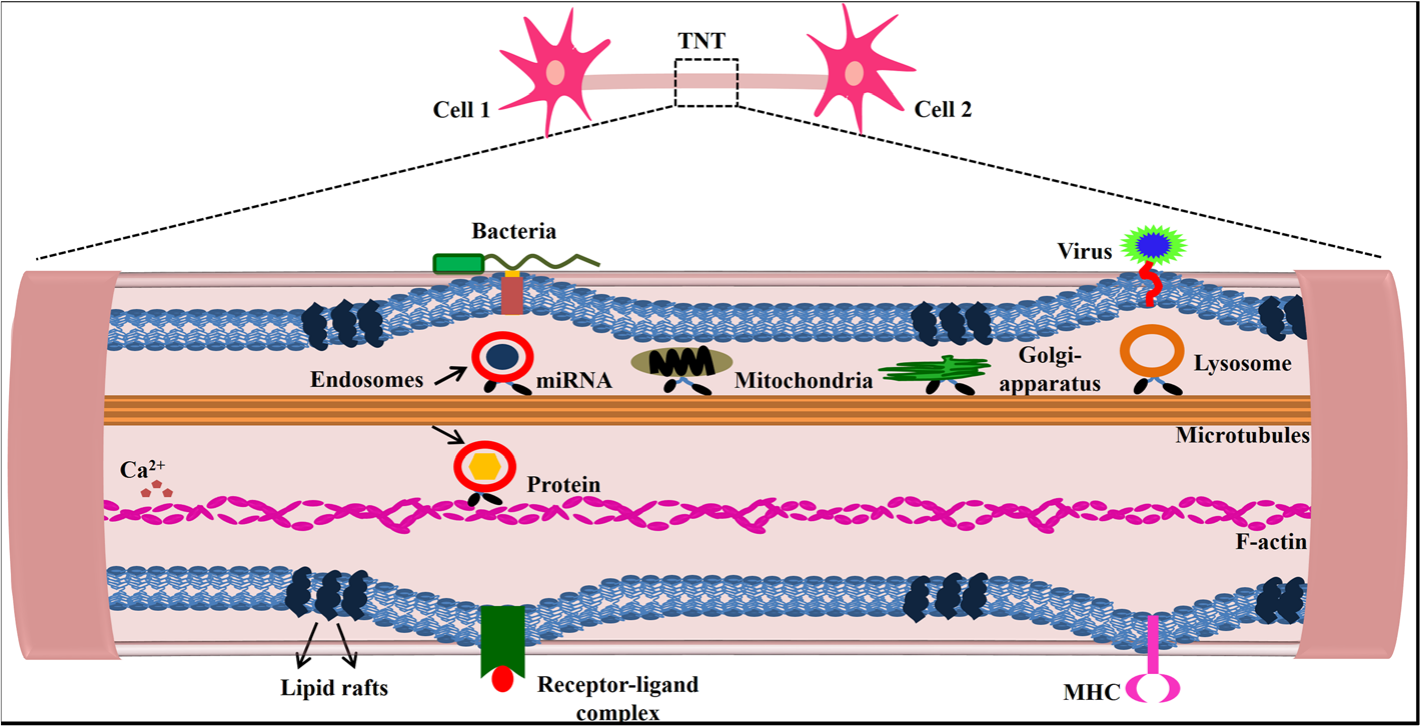
Formation of TNT’s for exchange or transfer of different cellular organelles such as mitochondria, Golgi apparatus, lysosomes. These are involved in exchange of important proteins, nucleic acids and ions associated in different signalling cascades. TNT’s express different receptors which are involved in bacterial and viral infection progression [15, 57–60]

Another study determined the role of nanotubes in early events associated with BCR signal transduction. They demonstrated the dynamic nature of TNTs and their ability to move laterally on the surface of the cell, which had been also characterized by early experiments on nanochannels in cancer cells. They also identified CD45 and Syk as molecular markers for induction of TNT formation in B cells as evidenced by their inducible co-localisation with the nanotubes [49]. A 2001 study also observed the formation of TNTs in human neutrophils in vitro when cultured in specific conditions, specifically requiring Na + free medium which inhibited the neutrophils from spreading. Thick nanochannels had unattached tips radiating out from the cell body, which grew up to 70 μm in just 20 mins. Upon addition of Na + ions, spreading was restored and nanochannel formation was reduced. TNT formation in neutrophils was observed to be an alternative mechanism for cell adhesion and communication in the study [50]. Figure 2 summarizes the cell-to-cell interaction mediated by nanochannel.

## Endothelial cells form TNTs for intercellular transport

TNTs are also known to play a significant role in maintaining the function of endothelial cells along with their surrounding tissues. Endothelial cells that are present in the extra-cellular matrix surrounding tumor tissue was found to form extensive TNTs with the tumor cells, and transfer of organelles between the different cell types modulate chemo-resistance in tumor cells, as well as endothelial phenotype [22, 30]. In a recent 2015 study [51], TNTs were found between microvascular endothelial cells (HMECs) that transported organelles such as mitochondria and lysosomes, as well as lipid droplets through membrane connections through distances as long as over 100 μm. VEGF treatment was seen to significantly increase the number of lipid droplets, while arachidonic acid not only increased lipid droplet transport but also induced a 3-fold increase in TNT formation [51].

Yasuda et al. also discovered the effect of direct mitochondrial and lysosomal transfer between endothelial cells through TNTs and identified it as the primary driving force behind robust functional improvement in vasculopathies beyond endothelial progenitor cells [52]. Endothelial progenitor cells (EPCs) were observed to form extensive TNT connections with stressed endothelial cells, and subsequent lysosomal transfer not only improved endothelial cell viability, but also reduced premature senescence and apoptosis. Human umbilical vein endothelial cells (HUVECs) were used in the study along with EPCs, and there was observed to be a 3 fold increase in organelle transfer when the HUVEC cells were exposed to increased concentrations of cytotoxic drugs, indicating TNTs to be a specific pathway for repair [52]. Another study on the neuroprotective properties of neural stem cells (NSCs) also demonstrated a key role of TNT formation between NSCs and the brain microvascular endothelial cells (BMECs). Stressed BMECs from sites of ischemic injury were observed to induce TNT formation in surrounding NSCs in in vitro conditions. The TNTs were composed of both F-actin and microtubules, and electron microscopy was used to demonstrate mitochondrial exchange [53]. M-Sec, a marker for TNT formation, was observed to enhance the formation of TNTs between cells when up-regulated and was proposed as a possible therapeutic target for ischemic stroke injuries [53]. M-Sec is shown to be the marker for TNT and play important role in its formation. M-sec can induce the formation of TNTs in co-operation with RalA small GTPase and the exocyst complex which suggest the role actin remodeling in TNTs formation [54]. M-Sec gene expression is induced by TNF-alpha. Retinoic acid is also shown to induce this gene expression. Overexpression of TNFAIP2 (M-Sec) reverses the inflammatory response and miR-221 induced apoptosis. RNAi based therapy can be utilized for targeting TNT structure. Other than RNAi based approach, actin inhibitor such as Cytochalasin can be utilized for targeting TNT formation. Drugs such as Nocodozole (Microtubule) can be used for inhibiting growth of TNTs.

## Conclusion

TNTs are one of the most novel methods of mechanical intercellular communication over long distances, and research over the last two decades has demonstrated TNTs to be increasingly ubiquitous among different cell types. TNTs are used by cells for intercellular communication through calcium fluxes, proteins, miRNAs and organelles such as mitochondria, endosomes, and lysosomes. These components are transported through nanochannels along F-actin or microtubules by motor proteins, and hence require ATP. TNTs between immune cells, endothelial cells and stem cells are used to reverse stress and diseased conditions through intercellular mitochondrial and lysosomal transfer [52, 53]. TNTs also play a very important role in diseased conditions such as cancer. Nanotubes aid tumor cells in proliferation, as well as migration and initiation of metastasis [1, 22]. TNTs between cancer cells and cells from the surrounding extra-cellular matrix facilitate transfer oncogenic mRNAs to healthy cells [26]. TNTs between cancer cells also allows them to rapidly modulate the chemo-resistance of their neighbouring tumor cells [26, 28, 30]. All of these functions are affected when cells were treated with drugs that inhibit TNTs. Cytoskeletal inhibitors such as Latrunculin A, Cytochalasin D, Docetaxal [22], Jasplakinolide [39] as well as mTOR signalling inhibitors such as Metformin and Everolimus [29] are used as pharmacological inhibitors for nanotube formation. Markers such as M-Sec can also be used for targeted therapies [53]. Proteins that are involved in receptor-dependent HIV transmission between immune cells such as Gag [2], Env and CD4 [37] are also effective targets for peptide or pharmacological inhibitors. The cellular signals and machinery involved in TNT formation and maintenance in disease conditions can therefore be specifically targeted for novel therapies for cancers, as well as viral diseases such as AIDS [2, 37], Herpes [38], chickenpox (***varicella***) and porcine pseudorabies [39]. Cellular machinery such as actin remodeling, inflammatory signals and microtubule organization can be targeted to inhibit the formation of TNTs. Targeting molecules such as M-sec along with miR221 can be manipulated to target the formation of TNTs. As cancer cells secretes several inflammatory cytokines such as TNF-alpha and TNF-alpha is one of the important factor associated with M-sec gene expression, inhibiting expression of TNF-alpha can block the formation of TNTs and hence intercellular communication between cancer cells. Viruses escape the recognition by immune cells by spreading through TNTs. As the immunological synapse involved in TNT formation, TNT growth can be targeted by targeting these synapse. Combination therapies for Influenza both targeting TNT formation and neuraminidase have already been established as effective strategies in preclinical trials.

With the growing knowledge about TNTs, it is now well known that they are involved in different functions which includes cellular communication to disease progression. However, due to their recent discovery, there are many aspects which still need to be explored. As discussed previously, TNT’s are formed through actin driven de novo process, however, there are certain alternative mechanism also through which they are formed. Detailed knowledge about these alternative and de novo mechanisms which includes “what triggers them to form” especially in case of cancer cells can proved to be useful from therapy point of view. Additionally, they are known to be involved in microbial and viral infection. Detailed overview of all the receptors present on them can provide much deeper insight to the mechanism of disease progression. Further, therapeutic resistance which has been linked with TNT’s can also be explored thoroughly in order to circumvent it. One of the major challenge in this field is presence of different types of TNTs which are cell specific. Transcriptomics and proteomics based studies can further give us more insight about these nano-level interaction. Finally, there is need to explore the role of TNTs in connection with reactive oxygen species (ROS) which may further help in finding their role in complex diseases such as diabetes, Alzheimer’s and Parkinson’s.

## Abbreviations

ATP: Adenosine triphosphate
BCR: B cell receptor
CD4: Cluster of differentiation 4
CD45: Cluster of differentiation 45
EBV: Epstein-Barr Virus
Env: Envelope protein
EPC: Endothelial progenitor cell
Gag: Group-specific antigen
GFP: Green fluorescent protein
HIV: Human immunodeficiency virus
MHC: Major histocompatibility complex
miRNA: Micro RNA
mTOR: Mechanistic target of rapamycin
NK cells: Natural Killer cells
SEM: Scanning electron microscope
SYK: Spleen associated tyrosine kinase
TEM: Transmission electron microscope
TNTs: Tunneling nanotube
VEGF: Vascular endothelial growth factor
VZV: Varicella-zoster virus
